# Effect of Calorie Restriction and Intermittent Fasting Regimens on Brain-Derived Neurotrophic Factor Levels and Cognitive Function in Humans: A Systematic Review

**DOI:** 10.3390/medicina60010191

**Published:** 2024-01-22

**Authors:** Refat Alkurd, Lana Mahrous, Falak Zeb, Moien AB Khan, Hamid Alhaj, Husam M. Khraiwesh, MoezAlIslam E. Faris

**Affiliations:** 1 Department of Nutrition, Faculty of Pharmacy and Medical Sciences, University of Petra, Amman 11196, Jordan; ralkurd@uop.edu.jo; 2 Department of Health Sciences/Track of Clinical Nutrition, College of Health and Rehabilitation, Princess Nourah Bint Abdulrahman University, Riyadh 12461, Saudi Arabia; lana.mahrous@gmail.com; 3 Nutrition and Food Research Group, Research Institute of Medical and Health Sciences (RIMHS), University of Sharjah, Sharjah 27272, United Arab Emirates; fzeb@sharjah.ac.ae; 4 Health and Wellness Research Group, Department of Family Medicine, College of Medicine and Health Sciences, United Arab Emirates University, Al Ain 15551, United Arab Emirates; moien.khan@uaeu.ac.ae; 5 Family and Community Medicine and Behavioral Sciences, College of Medicine, University of Sharjah, Sharjah 27272, United Arab Emirates; halhaj@sharjah.ac.ae; 6 Department of Nutrition and Food Processing, Faculty of Agricultural Technology, Al-Balqa Applied University, Salt 19117, Jordan; khraiwesh@bau.edu.jo; 7 Department of Clinical Nutrition and Dietetics, College of Health Sciences, University of Sharjah, Sharjah 27272, United Arab Emirates; 8 Healthy Aging, Longevity and Sustainability Research Group, Research Institute of Medical and Health Sciences (RIMHS), University of Sharjah, Sharjah 27272, United Arab Emirates

**Keywords:** intermittent fasting, BDNF, cognitive function, Ramadan fasting, metabolic condition

## Abstract

*Background:* The potential positive interaction between intermittent fasting (IF) and brain-derived neurotrophic factor (BDNF) on cognitive function has been widely discussed. This systematic review tried to assess the efficacy of interventions with different IF regimens on BDNF levels and their association with cognitive functions in humans. Interventions with different forms of IF such as caloric restriction (CR), alternate-day fasting (ADF), time-restricted eating (TRE), and the Ramadan model of intermittent fasting (RIF) were targeted. *Methods:* A systematic review was conducted for experimental and observational studies on healthy people and patients with diseases published in EMBASE, Scopus, PubMed, and Google Scholar databases from January 2000 to December 2023. We followed the Preferred Reporting Items for Systematic Reviews and Meta-Analysis statements (PRISMA) for writing this review. *Results:* Sixteen research works conducted on healthy people and patients with metabolic disorders met the inclusion criteria for this systematic review. Five studies showed a significant increase in BDNF after the intervention, while five studies reported a significant decrease in BDNF levels, and the other six studies showed no significant changes in BDNF levels due to IF regimens. Moreover, five studies examined the RIF protocol, of which, three studies showed a significant reduction, while two showed a significant increase in BDNF levels, along with an improvement in cognitive function after RIF. *Conclusions:* The current findings suggest that IF has varying effects on BDNF levels and cognitive functions in healthy, overweight/obese individuals and patients with metabolic conditions. However, few human studies have shown that IF increases BDNF levels, with controversial results. In humans, IF has yet to be fully investigated in terms of its long-term effect on BDNF and cognitive functions. Large-scale, well-controlled studies with high-quality data are warranted to elucidate the impact of the IF regimens on BDNF levels and cognitive functions.

## 1. Introduction

Brain-derived neurotrophic factor (BDNF) is a protein (neurotrophin) that is mainly produced in the central nervous system. Its function depends on the stage of brain development and it is involved in processes such as synaptic transmission and synaptic plasticity, which contribute to cognitive function [1,2]. Furthermore, BDNF modulates metabolism and controls eating patterns and food intake behaviors as well as contributing to energy homeostasis [3]. In addition, in a zebrafish model, BDNF was found to affect physical performance and glucose metabolism, which may influence food appetite, insulin sensitivity, and parasympathetic cardiovascular tone [4]. Previous studies have shown that lower BDNF concentrations are associated with cognitive impairment, obesity, and metabolic syndrome, while higher BDNF concentrations are associated with improved cognitive performance and metabolic health [5,6,7]. As a result of its effect on glucose oxidation and food intake, BDNF may lower blood glucose levels and increase insulin sensitivity [8]. Healthy diet and lifestyle behaviors, such as physical activity, are well known to preserve cognitive function and metabolic health [9].

Fasting has been advocated as one of the candidate therapies for neurological disorders. This comes by virtue of the fasting effect in improving cognition, slowing down neurodegeneration, reducing brain damage, enhancing functional recovery after stroke, and mitigating the pathological and clinical features of epilepsy and multiple sclerosis in animal models [10]. Among the emerging healthy diets, numerous studies have demonstrated that intermittent fasting (IF) has significant effects on weight changes and metabolic parameters associated with type 2 diabetes, cardiovascular disease, oxidative stress, and cancer [11,12,13,14]. Furthermore, many animal studies have shown that IF reduces cognitive deficits by stimulating a reduction in BDNF production in the hippocampus, cerebral cortex, and striatum by suppressing the expression of proinflammatory cytokines, such as IL-1β, and enhancing neurotrophic support [15,16,17,18].

Intriguingly, recent research unraveled that as humans age, the brain experiences a decline in neurogenesis and synaptic plasticity, contributing to cognitive decline. However, BDNF was found to improve brain function by promoting both neurogenesis and synaptic plasticity, particularly through a process called long-term potentiation (LTP), a process involving persistent strengthening of synapses that leads to a long-lasting increase in signal transmission between neurons [2]. This is achieved by BDNF acting directly binding to a receptor called tropomyosin receptor kinase B, also known as tyrosine receptor kinase B (trkB). This BDNF/TrkB signaling pathway supports neuronal survival, plasticity, differentiation, and growth via the activation of several functional downstream cascades, and ends with triggering both pre-and postsynaptic changes that enhance communication between brain cells [19]. Interestingly, IF is a potent inducer of BDNF signaling, along with an adaptive stress response. This upregulates protein synthesis and further boosts neuroplasticity, leading to improved learning and memory. Therefore, understanding the interplay between brain aging, BDNF, and IF will open exciting avenues for promoting cognitive health and potentially mitigating age-related cognitive decline [20].

A previous review on athletes at rest and during exercise demonstrated that BDNF signaling in the brain can affect some behavioral and metabolic reactions in response to IF, including exercise and activity levels, appetite regulation, cognitive development, and glucose metabolism [21]. However, few human studies have shown the effect of IF on BDNF production. In contrast, a solicited study examining the effects of IF and long-term food restriction on BDNF in human subjects pointed toward a negative impact of IF and long-term food restriction on cognitive performance. To the best of our knowledge, no published systematic review has exclusively examined the effectiveness of interventions with IF on BDNF levels and the associated changes in cognitive functions in human subjects. In this structured systematic review, the main aim was to investigate the effect of interventions with different IF regimens on the concentration of BDNF in human subjects as well as to examine the impact of IF on cognitive functions through the BDNF pathway.

## 2. Materials and Methods

### 2.1. Search Strategy

To identify the available studies, a detailed search relating to CR, IF, and BDNF was conducted according to the PRISMA guidelines [22]. A systematic literature search was performed in the electronic databases EMBASE, Scopus, PubMed, and Google Scholar, using the following search terms in all possible combinations: intermittent fasting OR calorie restriction AND brain-derived neurotrophic factor OR BDNF AND cognitive function OR mental health. Additionally, a manual search was conducted through the reference lists of all collected articles to ensure that all relevant studies were identified and to avoid any missing relevant data.

### 2.2. Data Extraction and Quality Assessment

#### 2.2.1. Study Selection and Data Extraction

**Figure 1:**
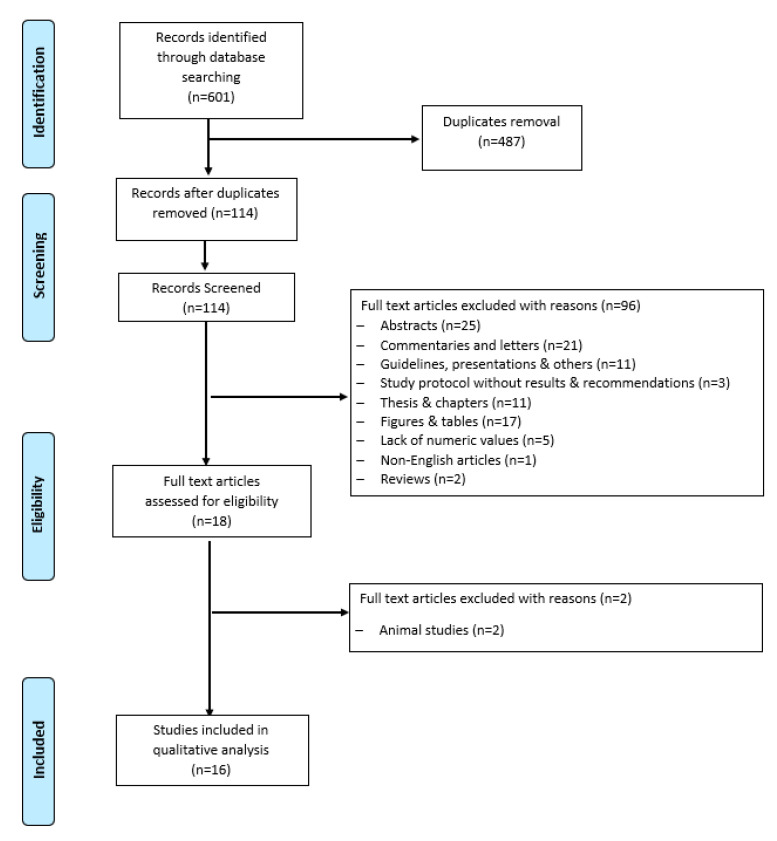
Flow diagram of literature search and study selection.

All clinical studies evaluating the impact of IF and CR on BDNF levels in humans were reviewed and read carefully to identify their relevancy. To identify the eligible studies, the titles and abstracts were screened in the first phase of the selection procedure by two independent researchers (LM, MF). In the second phase, the full articles were screened for eligibility. The eligible criteria for the inclusion of the articles in this review were clinical trials, observational studies, and correlations between serum/plasma BDNF levels and IF and CR in healthy individuals or individuals with comorbidities. Studies conducted in animal models, case reports, reviews, and duplicate studies were excluded. We extracted items for the characteristics of the articles including the first author, publication year, study design, disease condition, sample size with male/female ratio, IF protocol applied, duration, exercise intervention, assessment of BDNF levels, and cognitive function tested. A flow diagram of the literature search and selection is shown in Figure 1. Potentially relevant studies (*n* = 601) were identified by searching electronic databases. Duplicates were removed and those studies that only included humans were selected.

#### 2.2.2. Assessment of the Quality of Studies

To assess the quality of the included studies, the Preferred Reporting Items for Systematic Reviews and Meta-Analyses checklist was used. We used a tool recommended by the Cochrane Collaboration for assessing the selection bias, performance bias, detection bias, attribution bias, and reporting bias of the included studies [23].

## 3. Results

The characteristics of the included studies are summarized in Table 1. The sixteen studies were conducted between 2007 and 2023. Two of the studies [24,25] were conducted on patients with schizophrenia, while the rest were conducted on people without a psychological disorder, two of which were conducted on people diagnosed with metabolic syndrome [26,27]. Nine of the sixteen studies were experimental, while the other seven studies were observational. Five of the sixteen studies were executed during the month of Ramadan [25,26,28,29,30]. Three of the five RIF studies revealed reductions in BDNF levels [25,26,30], while the rest (two studies) revealed an increase in BDNF levels during/after the observance of RIF [28,29]. Among the sixteen selected studies, eleven studies did not involve calorie restriction [15,25,26,27,28,29,30,31,32,33,34], while the other five studies did [24,35,36,37,38]. Six studies were conducted in the USA [15,26,27,31,33,36], four in Germany [29,30,32,37], and one in each of the following countries: Egypt [25], UK [35], Brazil [24], New Zealand [34], Iran [28], and Denmark [38].

In this review, BDNF was the primary outcome of interest in association with IF in human studies. In total, 36 relevant publications were found using the literature search. However, 16 studies were experimental studies examining the effect of different types of IF including CR, TRE, RIF, and ADF on BNDF levels in healthy individuals; patients with metabolic syndrome or a neurodegenerative disorder; and overweight or obese individuals. Overall, six of the included studies showed no significant changes in the serum BNDF concentration after the IF intervention. Five studies showed a significant increase and the other five showed a significant decrease in BDNF concentration. Nevertheless, five out of the sixteen studies used the RIF protocol, and by observing RIF, we found that three studies showed significant decreases and two studies showed significant increases in BDNF levels. In addition, only four studies showed that IF therapy may positively influence cognitive function while the rest did not assess cognitive performance.

Interestingly, the results of Catenacci et al. [36] suggested that ADF induced significant changes in BDNF secretion at the 24-week follow up and this alteration may be due to weight loss as BDNF can play a role in the regulation of energy balance that ultimately reduces adiposity. However, this result should be carefully construed, as changes in BDNF may correlate with changes in weight or body composition. Similarly, Bastani et al. [28] observed a significant increase in plasma BDNF on the 14th day (second group) and 29th day (third group) of RIF compared to pre-Ramadan levels. The BDNF level in the second group was increased significantly by 25% and by 47% in the third group compared to the control group (*p* < 0.05). Another study suggested that TRE may have a direct impact on the central circadian clock and has the tendency to affect hormonal levels depending on meal timing. They also showed that TRE reduced cortisol levels by 1.4 ± 0.6 μg/dL (*p* = 0.03) and tended to increase BDNF levels by 2.46 ± 1.34 ng/mL (*p* = 0.09) in the evening [15]. BDNF is a well-recognized protein for the regulation and adaptation of energy balance at the cellular level [39]. However, many lifestyle interventions like IF exert changes in energy balance that may influence the level of BDNF.

A study demonstrated that RIF significantly increases the BDNF level at the end of Ramadan; however, the BDNF level decreased and returned to the baseline value one week post Ramadan [29]. This could be explained by the fact that BDNF can be altered in the energy balance adaptation process. Similarly, the CR diet also induced an increase in BDNF levels in schizophrenic patients and it was suggested that the improvement in dietary nutrients and food quantity may modify important markers of brain plasticity. However, the BDNF level may also increase in patients taking high antipsychotics daily (*r* = 0.216; *p* = 0.098), which also typically increases BDNF levels [24].

In contrast, we found that five studies showed a significant reduction in BDNF levels after IF. Similarly, a study found a significant reduction in BDNF levels while demonstrating significant improvement in a patient with focal seizures post-Ramadan. Thus, it was proposed that RIF plays a role in inducing the transcription of BDNF which stimulates the production and survival of new hippocampal neurons, maintains the synaptic structure, and thus promotes more sustained neuronal resistance to stress [40]. Fawzi et al. [25] found that the change in total energy and BMI during Ramadan fasting were significant and independent variables associated with the increase in serum BDNF levels by 44%; however, they could not demonstrate any benefits in schizophrenia patients as a lower BDNF level may worsen the psychiatric status, such as a relapse of bipolar disorder. Moreover, previous studies also reported a significant decrease in BDNF levels upon IF intervention [30,35,38]; however, the results indicated that other factors may contribute to BDNF alterations like body composition parameters, hormonal status, sex, and exercise. On the other hand, a study reported that intermittent energy restriction induces a positive mood when the cognitive function in overweight individuals was assessed and this effect was due to increased self-confidence and ongoing motivational calls in their 6-month weight loss journey rather than the alteration in BDNF concentrations [35]. In addition, other studies suggested that RIF may positively affect cognitive function by improving the individual’s mood along with a significant change in the BDNF level at the end of the Ramadan fasting month [28,30]. The reported partial improvement in BDNF levels after observing RIF could be also ascribed to the effect of RIF in reducing body weight [41], adiposity [42], visceral adiposity [43], metabolic syndrome components [44], cardiometabolic risk factors [45], proinflammatory cytokines and oxidative stress markers [46,47], and IGF-1 [43]; all of these have been implicated in the pathogenesis of mental health problems and decreased BDNF levels.

## 4. Quality Assessment

The systematic review included a diverse range of study types, each contributing unique insights. The randomized controlled trials (RCTs), comprising Jamshed et al. [15], Carlson et al. [31], Harvie et al. [35], Catenacci et al. [36], Bastani et al. [28], Schübel et al. [37], Glud et al. [38], Wallace et al. [33], and Bartholomew et al. [27], offered rigorous experimental data. Complementing these were non-RCTs like Kessler et al. [32] and Abdulsada et al. [26]; prospective studies by Fawzi et al. [25] and Riat et al. [30]; a cross-sectional study by Guimarães et al. [24]; a prospective controlled trial by Ghashang et al. [29]; and a study using a repeated measures cross-over design by Gibbons et al. [34].

**Figure 2:**
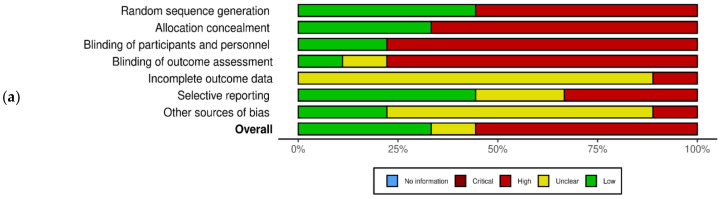

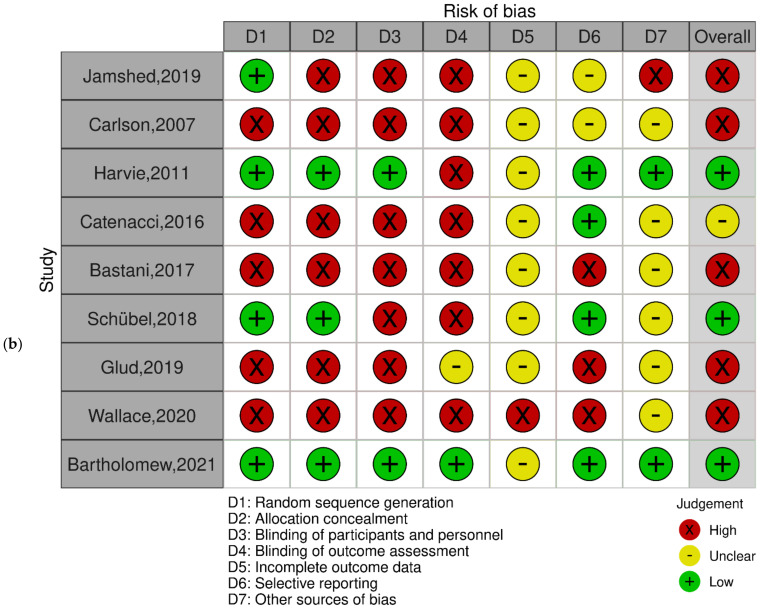
Quality assessment of the experimental studies included in the systematic review using the Cochrane tool. (**a**) Risk of bias with each risk of bias item for each included study; (**b**) risk of bias graph with each risk of bias item presented as percentages across all included studies. Green: low risk of bias; yellow: unclear risk of bias; red: high risk of bias (*n* = 9) [15,27,28,31,33,35,36,37,38].

The quality assessment of the studies using the Cochrane tool reveals varied results. Adequate sequence generation was observed in Jamshed et al. [15], mitigating selection bias. Allocation concealment was robust in Carlson et al. [31], enhancing the study’s internal validity. The blinding of participants, a critical aspect of reducing performance bias, was well-implemented by Catenacci et al. (2016) [36]. Attrition bias was minimized due to the comprehensive handling of incomplete outcome data by Bastani et al. [28]. However, the potential for detection bias was present, as outcome assessment blinding was not consistent across all studies, which was particularly noted in Harvie et al. (2011) [35]. The review also highlighted selective reporting and other biases in a subset of studies, including Schübel et al. [37], warranting cautious interpretation of these results (Figure 2a,b).

As per the Cochrane Risk of Bias Tool for Randomized Controlled Trials, the included studies had several sources of bias. We observed several forms of bias across the studies including selection bias, which was noted in the way participants were allocated to the intervention and control groups, potentially affecting the comparability of these groups; measurement bias, which pertained to inconsistencies in how outcomes were measured and recorded across different studies, which could influence the results; dietary adherence Bias, specific to studies involving dietary interventions, which arose from variations in participants’ adherence to dietary guidelines; time-frame bias, which was observed in the duration of the studies, which varied and might have impacted the outcomes; limited data points leading to bias, which refers to the scarcity of data points in some studies restricting our ability to draw comprehensive conclusions; lack of baseline measurement bias, which, in some studies, the absence of baseline measurements hindered the assessment of changes over time; and omission of relevant variables bias, which was noted in studies that failed to account for or report relevant variables that could influence the outcomes.

## 5. Discussion

This systematic review tried to assess the efficacy of interventions with different IF regimens on BDNF levels and their association with cognitive functions in humans. Interventions with different forms of fasting regimens such as CR, ADF, TRE, and RIF were targeted. The current results revealed mixed effects of CR and IF regimens on BDNF levels, with no clear picture being drawn concerning the effect of these dietary interventions on the targeted outcome, a matter that dictates the need for well-controlled, long-term experimental studies to elucidate the impact of IF and CR regimens on BDNF levels.

BDNF function is associated with energy metabolism and synaptic and behavioral plasticity, which influence the cognitive functions of learning and maintaining memory capacity in humans. Higher BDNF concentrations in the hippocampus have been associated with both improved cognitive function [48] and metabolic health [49]. Recently, many animal (rodent) studies have revealed that IF positively stimulates the production of BDNF in the hippocampus, cerebral cortex, and striatum and reduces cognitive deficits by enhancing neurotrophic support and suppressing the expression of pro-inflammatory cytokines [18,50,51].

In terms of human studies, the increase in BDNF concentrations due to IF interventions has attracted considerable attention. In this systematic review, we analyzed and evaluated 16 human intervention studies from the literature to investigate the effect of CR and different IF regimens on BDNF concentration and cognitive function. During the evaluation, several concerns resulted in complex results and we could not determine the effect of IF on BDNF concentrations and cognitive function due to the varied application of different methodologies in the studies. First, the collection of the peripheral BDNF concentration varied between studies which related to whether the BDNF concentration was measured from serum or plasma samples; in addition, the centrifuge protocol, clotting period, and temperature can affect the measurement of plasma or serum concentrations in the studies which made it difficult to generalize the findings of peripheral BDNF concentrations between the 16 studies.

In animal studies, it was determined that there is a higher concentration of BDNF in sera than in plasma [52]. Moreover, reports indicated a correlation between fasting and an increase in BDNF levels in overweight or obese subjects, which was associated with changes in body composition or fat percentages [38,53,54,55]. However, in this review, some of the study results varied and this is because other major factors impacted the BDNF concentration related to IF. For example, changes in energy balance and gene expression during long hours of fasting such as Ramadan affect the regulation of hormones, such as cortisol [56] and insulin-like growth factor (IGF-1) [43], which alter the concentration of BDNF [57], as well as a sex-based biological parameter, which affects serum BDNF levels [26].

Further, RIF has been associated with the overexpression of a set of genes (*TFAM, SOD2*, and *Nrf2*) which have been implicated in improving neuroplasticity and decreasing neuroinflammation [58]. These genes showed significantly increased expression at the end of the fasting month, increasing by 90.5%, 54.1%, and 411.5% for the three genes, respectively [59]. In addition, the expression of the fat mass and obesity-associated (*FTO*) gene has been found to affect hippocampal function and regulate BDNF processing, which helps to further explain the intricate relationship between RIF and BDNF. A recent gene expression study revealed that RIF was associated with an approximately 30% reduction in the levels of *FTO* gene expression in overweight and obese people observing the fasting month [60], which helps to explain the controversial effects of RIF and CR on BDNF.

A study reported a 25% higher serum BDNF level in women compared to men, confirming that circulating BDNF levels are sex-dependent [38]. Previous reports also showed that women tend to have a higher expression of BDNF in several brain regions [38,61] and they have higher circulating BDNF levels in the last phase of their menstrual cycle compared to the first phase [61]. These findings suggested that gonadal hormones could influence the estrogen-specific effect on circulating BDNF levels more in women than in men. Furthermore, the expression of BDNF at the protein and mRNA levels is responsive to exercise and has a positive influence on cognition function [62]. However, BDNF can only be altered depending on the intensity of exercise, age, sex, body mass, diet, and fitness level of the individual [63,64,65]. Additionally, IF in a specific population such as schizophrenia patients provided inconsistent results in BDNF concentration due to the type of diet, the effect of drug treatments, phenotypes, the intensity of symptoms, and the duration of schizophrenia [17]. Notably, no significant outcome was found in the association between IF and BDNF concentration. There is a positive confirmed link between long-term healthy lifestyle interventions, including a healthy diet, calorie restriction, physical activity, and quality of sleep, and beneficial effects on BDNF in the brain and that the elevation of BDNF can improve neurodegeneration in the nervous system [20,66].

Recently, IF has emerged as a multifaceted approach that influences long-chain fatty acid oxidation through the intricate interplay of gut microbiota changes and BDNF production [67,68,69]. This triad not only offers insights into the metabolic benefits of IF but also sheds light on the complex connections between the gut, the brain, and metabolic health. Further research is warranted to elucidate the precise mechanisms underlying these interactions, paving the way for personalized interventions that harness the potential of IF for optimizing metabolic function and overall well-being.

The observed differences in the effect of IF on BDNF could be also explained by the sex-specific differences in lipid metabolism [70]. In one clinical trial, sex was found to be one of the biological determinants that shaped the effect of fasting on circulating BDNF levels. Such a difference was also a mirror for the differences in the effect of observing RIF on the two sexes in healthy and disease conditions, as revealed by previous reviews [71,72].

Astroglia, a type of glial cell in the brain, play a crucial and multifaceted role in the metabolism of fatty acids, contributing significantly to overall brain function [73]. These star-shaped cells are not merely passive supporters; they actively participate in the intricate biochemical processes that sustain neural health [74]. Astroglia are instrumental in the uptake, storage, and utilization of fatty acids, serving as key intermediaries in the intricate lipid metabolism within the brain [75]. Through their sophisticated network of processes, astroglia contributes to energy homeostasis, neurotransmitter synthesis, and neuroprotection [76]. The close interaction between astroglia and neurons highlights the dynamic interplay between different cell types in the brain, emphasizing the intricate balance required for optimal cognitive function and overall neurological well-being [73,76]. Gaining knowledge about the involvement of astroglia in the metabolism of fatty acids offers valuable information on potential therapeutic approaches for different neurological disorders. It also enhances our understanding of the impact of IF on brain health, the suggested neuroprotective effects of IF, and the mediating role of BDNF in brain health and cognitive function.

In the context of studying the effect of IF on BDNF, the complex process of synaptic mitochondria efficiently oxidizing long-chain fatty acids gains particular relevance [77]. During fasting periods, the brain’s reliance on alternative energy sources, such as the oxidation of long-chain fatty acids by synaptic mitochondria, may enhance the production of ketone bodies and trigger a metabolic state that supports increased BDNF expression [50,78]. The nuanced interplay between fatty acid metabolism and BDNF in the context of IF highlights the intricate molecular mechanisms underlying the potential cognitive benefits associated with this dietary intervention [79], offering a promising avenue for further exploration in neuroscientific research.

Our study highlights several directions for future research. Reliable protocols for the assessment of peripheral BDNF levels need to be developed to enable a proper evaluation of the peripheral concentration of BDNF in future studies. More RCTs in humans are crucial to investigating the effectiveness of IF on cognitive function in obese or healthy individuals. In addition, the duration and type of IF intervention and incorporation of hormones or biomarker outcomes could help to estimate the effect of peripheral BDNF concentrations. It should be noted that this review has several limitations, including the presence of only 16 RCTs with a variety of methodologies. Secondly, the inclusion criteria varied, with some studies including healthy individuals and others excluding those with metabolic syndrome or neurological disorders. Thirdly, the type of fasting and diet in the IF interventions varied. Nevertheless, our study is the first systematic review to examine IF’s effect on BDNF concentrations and cognitive functions in humans. To identify the long-term effects of IF on BDNF, high-quality research with large-scale randomized controlled trials is required.

## 6. Conclusions

There are controversial results from human studies regarding the IF and CR effects on BDNF levels. The long-term effects of IF on BDNF levels have yet to be investigated. Due to the dissimilarity of the studies’ outcomes and contradictory findings, this review cannot generalize the results based on human trial interventions related to IF and BDNF levels. To understand the impact of IF regimens on BDNF levels and cognitive functions, large-scale, controlled clinical studies are greatly needed.

## Figures and Tables

**Table 1:** Characteristics of the included studies.

Reference/ Country	Study Design	Health Condition	Subject M/F Ratio	IF Strategy	Duration	Inclusion of Exercise	Effect on BDNF Level	Cognitive Performance	Involvement of Calorie Restriction
Carlson et al., 2007 [31]/USA	RCT	Healthy	5 M/10 F	1 meal per day (for 4 h in the early evening; 4:00 to 8:00 p.m.)	16 weeks	N/A	↔	N/A	No CR
Guimaraes et al., 2008 [24]/ Brazil	Cross-sectional	Schizophrenia	51 M/ 16 F	Hypo-caloric diet, CR	4 weeks	N/A	↑	N/A	CR: F 1600–2000 kcal/day; M 2000–2300 kcal/day
Harvie et al., 2010 [35]/UK	RCT	Overweight	89 F	IER	6 months	N/A	↔	Positive effect on mood	CR: 25% CR as IER (~2266 kJ/day for 2 days/week) or CER (~6276 kJ/day for 7 days/week) 2266 kJ = ~540 kcal; 6276 kJ = 1500 kcal
Fawzi et al., 2014 [25]/Egypt	RCT	Schizophrenia	100 M	RIF	4 weeks	N/A	↓	N/A	No CR; just RIF
Catenacci et al., 2016 [36]/USA	RCT	Obesity	6 M/19 F	ADF/CR	8 weeks	N/A	ADF ↑/CR ↓	N/A	Either zero-calorie ADF or CR (−400 kcal/day)
Bastani et al., 2017 [28]/Iran	RCT	Healthy	7 M/22 F	RIF	4 weeks	N/A	↑	Positive change in cognitive health	No CR; just RIF
Kessler et al., 2017 [32]/ Germany	Non-RCT	Healthy	22	1-day fasting/week	8 weeks	N/A	↔	N/A	No CR
Schübel et al., 2018 [37]/ Germany	RCT	Obesity	150 F	ICR and CCR	50 weeks	N/A	↔	N/A	Either ICR or CCR (daily energy deficit ∼20%)
Ghashang et al., 2019 [29]/ Germany	Prospective CT	Healthy	50 M	RIF	4 weeks	N/A	↑	N/A	No CR; just RIF
Glud et al., 2019 [38]/ Denmark	RCT	Overweight/ Obesity	24 M/ 26 F	CER	12 weeks	3× per week with 60–75 min	↓	N/A	Included very low-energy diet (VLED 600 kcal/day)
Jamshed et al., 2019 [15]/USA	RCT-crossover	Healthy and obesity	7 M/4 F	4 days of TRE 18 h	5 weeks	N/A	↑	N/A	No CR
Abdulsada et al., 2021 [26]/USA	RCT	Healthy and metabolic syndrome	21 M/7 F	RIF, 14 h fasting	4 weeks	N/A	↓	N/A	No CR; just RIF
Wallace et al., 2020 [33]/USA	RCT	Healthy	12 M	TRE (up to 16 h fasting window)	6 weeks	4x per week with a duration of 30–45 min	↔	N/A	No CR TRE with aerobic exercise (AE) or without AE
Riat et al., 2021 [30]/ Germany	RCT	Healthy	19 M/ 15 F	RIF	4 weeks	N/A	↓	Improvement in mood	No CR; just RIF
Bartholomew et al., 2021 [27]/USA	RCT	Metabolic syndrome	34 M/ 69 F	5:2 IF	6 months	N/A	↔	N/A	No CR
Gibbons et al., 2023 [34]/New Zealand	Crossover	Healthy	6 M/6 F	20 h fasting	Once	90 min light exercise, and high-intensity exercise	IF ↔	N/A	No CR

M/F = male/female ratio, RCT = randomized control trial, N/A = not applicable, IER = intermittent energy restriction, ADF = alternate-day fasting, CR = caloric restriction, ICR = intermittent caloric restriction, CCR = continuous caloric restriction, ↔ = no significance, ↑ = significant increase, ↓ = significant decrease, CER = continuous energy restriction (600–800 kcal per day), TRE = time-restricted eating.

## Data Availability

The raw data supporting the conclusions of this article will be made available by the authors on request.
